# Transcriptional Mechanisms of Thermal Acclimation in *Prochlorococcus*

**DOI:** 10.1128/mbio.03425-22

**Published:** 2023-04-13

**Authors:** Laura Alonso-Sáez, Antonio S. Palacio, Ana M. Cabello, Semidán Robaina-Estévez, José M. González, Laurence Garczarek, Ángel López-Urrutia

**Affiliations:** a AZTI, Marine Research, Basque Research and Technology Alliance (BRTA), Sukarrieta, Spain; b Department of Microbiology, University of La Laguna, La Laguna, Spain; c Sorbonne Université, CNRS, UMR 7144 Adaptation and Diversity in the Marine Environment (AD2M), Station Biologique de Roscoff (SBR), Roscoff, France; d Centro Oceanográfico de Gijón, Instituto Español de Oceanografía, IEO-CSIC, Gijón, Asturias, Spain; University of California, Irvine

**Keywords:** marine, *Prochlorococcus*, thermal acclimation, transcriptomics, cyanobacteria

## Abstract

Low temperature limits the growth and the distribution of the key oceanic primary producer *Prochlorococcus*, which does not proliferate above a latitude of ca. 40°. Yet, the molecular basis of thermal acclimation in this cyanobacterium remains unexplored. We analyzed the transcriptional response of the Prochlorococcus marinus strain MIT9301 in long-term acclimations and in natural *Prochlorococcus* populations along a temperature range enabling its growth (17 to 30°C). MIT9301 upregulated mechanisms of the global stress response at the temperature minimum (17°C) but maintained the expression levels of genes involved in essential metabolic pathways (e.g., ATP synthesis and carbon fixation) along the whole thermal niche. Notably, the declining growth of MIT9301 from the optimum to the minimum temperature was coincident with a transcriptional suppression of the photosynthetic apparatus and a dampening of its circadian expression patterns, indicating a loss in their regulatory capacity under cold conditions. Under warm conditions, the cellular transcript inventory of MIT9301 was strongly streamlined, which may also induce regulatory imbalances due to stochasticity in gene expression. The daytime transcriptional suppression of photosynthetic genes at low temperature was also observed in metatranscriptomic reads mapping to MIT9301 across the global ocean, implying that this molecular mechanism may be associated with the restricted distribution of *Prochlorococcus* to temperate zones.

## INTRODUCTION

*Prochlorococcus* is the most abundant photosynthetic organism on Earth (1) and a major contributor to oceanic primary production (2). Despite sustaining vast populations at the global scale, *Prochlorococcus* exhibits an enigmatic distribution in the ocean, with a sharp latitudinal barrier at ca. 45°N and 40°S (3, 4). While the ultimate reasons for such restricted distribution are still unknown and may involve biotic interactions (5), temperatures in the range of 12 to 15°C typically limit the growth of *Prochlorococcus* in culture (3, 6, 7) and *in situ* (3), potentially representing critical thresholds for the metabolism of this cyanobacterium. Due to genome streamlining, *Prochlorococcus* is assumed to have a lower regulatory capacity than other phytoplankton groups and a limited metabolic flexibility to adapt to environmental disturbance, including temperature changes (8–10). However, a mechanistic understanding of *Prochlorococcus*’ temperature sensitivity at the molecular level is still lacking.

In general, addressing the impact of temperature on the functioning of any organism is complex, as this parameter has overriding effects on virtually every aspect of cell physiology. Temperature notably impacts the cellular size, stability, and conformation of macromolecules and the kinetics of biochemical reactions, altogether leading to differences in cell growth (11). In the case of photosynthetic organisms such as *Prochlorococcus*, growth is closely linked to the photosynthetic capacity, which is also impacted by temperature (12–14). Under temperature conditions below the optimum, the slowdown in carbon fixation rates constrains the replenishment of terminal acceptors of the photosynthetic electron flow, producing an imbalance between photochemistry and metabolism. Under these conditions, the excess of light energy absorbed can generate cell-damaging reactive oxygen species (ROS), which need to be counterbalanced by photoprotective mechanisms (15). Under heat stress conditions (35 to 50°C), ROS are also typically produced, likely not as a result of an excess of energy absorbed, but as a result of heat-induced structural and functional changes in the photosystems and thylakoid membranes (16). The decline in photosynthetic activity in phototrophs under moderate warm stress has been associated with different processes, including the inhibition of *de novo* synthesis of the photosystem II D1 protein by ROS (17), the inactivation of the oxygen-evolving complex (18), and declines in electron transport (19).

At the expression level, a variety of compensatory mechanisms have been found to be activated in several phytoplankton groups (i.e., *Synechococcus* and eukaryotic phytoplankton) to preserve cell functioning under thermal stress conditions (12–14, 20–23). The cold stress response network involves the upregulation of fatty acid desaturases to offset decreases in membrane fluidity at low temperature and RNA helicases and cellular chaperones to facilitate proper folding of nucleic acids and proteins (21, 22, 24, 25). Low temperature can also impact the expression of central components of the transcriptional and translational machinery in eukaryotic phytoplankton, which are upregulated to compensate for their reduced efficiency (21, 26), and even impact global regulatory networks, such as circadian rhythms in cyanobacteria (27). At elevated temperatures, a nearly universal response induces the expression of heat shock proteins, which degrade or restructure denatured proteins and nucleic acids (28).

Some of the temperature compensatory mechanisms involve only short-term transcriptional responses until cell functioning is restored (25). In other cases, the baseline expression of key enzymes is upregulated under long-term cold acclimations (26). Beyond short-term temperature manipulation experiments, where cells are suddenly exposed to a “thermal shock,” understanding mechanisms of long-term acclimation is particularly relevant as they more accurately reflect responses to gradually changing thermal conditions. Here, we performed a series of experiments on Prochlorococcus marinus MIT9301, where cells were progressively exposed from a temperature close to their optimum to their upper and lower thresholds of growth. This strain, which is a representative of the *Prochlorococcus* dominant clade *in situ* (HLII) (29, 30), was selected because it shares the highest sequence similarity with environmental sequences (31) and enabled the identification of closely related transcripts in natural *Prochlorococcus* populations. We first used a quantitative transcriptome sequencing (RNA-Seq) approach (32, 33) to decipher the transcriptional response of MIT9301 to temperature acclimations. Then, we addressed the environmental relevance of one of the most conspicuous transcriptional responses observed in MIT9301, involving highly expressed photosynthetic genes, in oceanic metatranscriptomes collected along a comparable thermal gradient. Our objective was to analyze how sustained growth at suboptimal temperature reprograms the transcriptome of *Prochlorococcus* and significantly advance our understanding of which mechanisms underlie the organism’s growth restriction under cold and warm conditions.

## RESULTS

### Thermal acclimation experiments with Prochlorococcus marinus MIT9301: growth rates and mRNA content.

**FIG 1:**
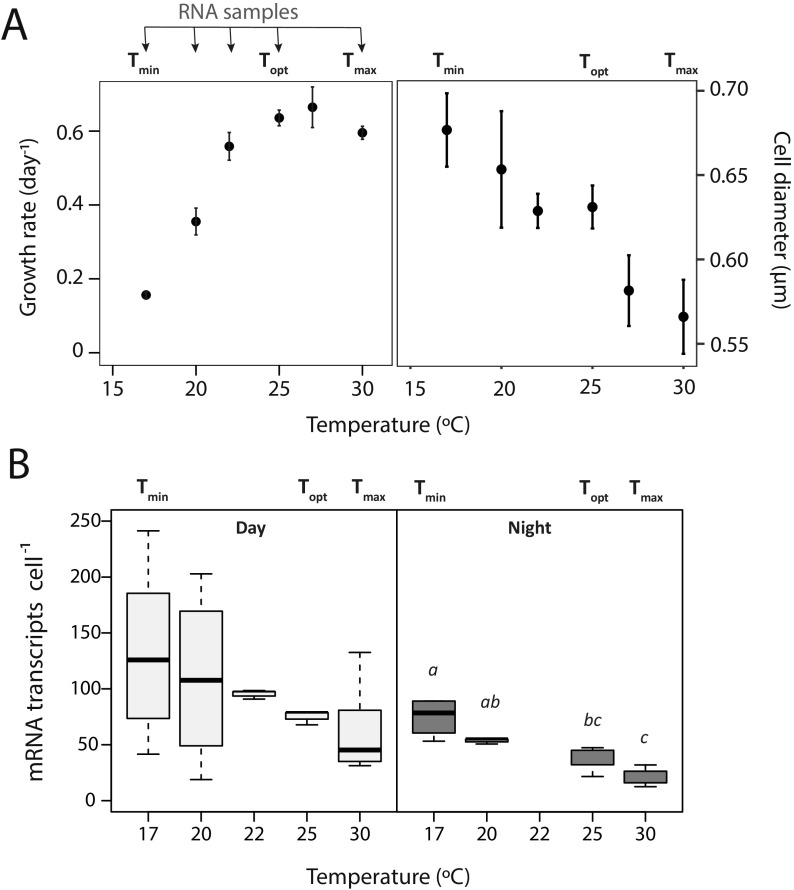
Growth rates, cell size, and number of mRNA transcripts per cell in samples of Prochlorococcus marinus MIT9301 collected after long-term thermal acclimation. Between 3 and 7 biological replicate samples are represented, depending on the temperature treatment, and error bars report the standard deviation between replicates. On the top of each plot, the minimum (T_min_), optimum (T_opt_), and maximum (T_max_) temperatures are indicated, and additionally, in the upper left plot, temperature treatments where RNA samples were collected are shown with arrows. (A) Growth rate and size of MIT9301 cells along the thermal niche. (B) Estimates of mRNA transcripts per cell along the thermal niche in samples collected at daytime (left panel) and nighttime (right panel). Lowercase italic letters denote statistically significant differences (analysis of variance [ANOVA] and Tukey *post hoc* test; *P* < 0.001).

MIT9301 cultures were synchronized to a diel 12-h/12-h light/dark cycle and long-term acclimated to six temperatures along the organism’s thermal range (Fig. 1; see Fig. S1 in the supplemental material). MIT9301 could not survive single-generation transfers below 17°C or above 30°C, and thus, these were considered minimum and maximum temperature thermal thresholds for this strain (here, T_min_ and T_max_, respectively). The growth rate of MIT9301 increased from 0.17 day^−1^ at T_min_ to ca. 0.61 day^−1^ at 25°C (here referred to as the optimum growth temperature [T_opt_]) and thereafter entered a warm stress zone up to 30°C, where no further increases in growth rate were observed (Fig. 1A). The average size of MIT9301 cells also changed along the thermal gradient, with maximum and minimum values at the T_min_ and T_max_, respectively (Fig. 1A).

10.1128/mbio.03425-22.1 FIG S1 Schematic diagram showing the experimental approach and sampling strategy during the long-term thermal acclimation experiments. While only 3 biological replicates are shown, the real number of replicates varied between 3 and 7, depending on the temperature treatment. Download FIG S1, PDF file, 0.3 MB. Copyright © 2023 Alonso-Sáez et al. 2023 Alonso-Sáez et al. https://creativecommons.org/licenses/by/4.0/ This content is distributed under the terms of the Creative Commons Attribution 4.0 International license.

After the acclimation period, RNA samples were collected under five different temperature conditions 3 h after subjective sunrise and sunset (daytime and nighttime, respectively) (see Table S1 in the supplemental material). Large variations in the number of mRNA transcripts per cell were found in replicate cultures acclimated to 17 and 20°C during daytime, while values were highly constrained in temperatures close to the optimum (22 and 25°C) (Fig. 1B). A pattern of decrease of mRNA transcripts with increasing temperature was found, with the average number of mRNA transcripts per cell being positively correlated with cellular size in nighttime samples (Spearman ρ = 0.89; *P* < 0.001).

10.1128/mbio.03425-22.4 TABLE S1 Basic information on biological replicate samples collected during the long-term thermal acclimation experiments with the isolate Prochlorococcus marinus MIT9301 after full acclimation to the temperature treatments. The parameters shown include the average recovery ratio of internal RNA standards in the samples and the cellular abundance and biovolume in the RNA filters collected. Download Table S1, XLSX file, 0.01 MB. Copyright © 2023 Alonso-Sáez et al. 2023 Alonso-Sáez et al. https://creativecommons.org/licenses/by/4.0/ This content is distributed under the terms of the Creative Commons Attribution 4.0 International license.

### Quantitative global transcriptomic analysis of MIT9301 in experimental long-term acclimations.

The top-expressed genes in MIT9301 were associated with photosynthetic components (e.g., *psbA* and *psbC*), the RuBisCO enzyme (*rbcL*) or an ammonium transporter (*amt*), which reached average values above two mRNA transcripts per cell (Table S2 and Table S3). However, the average abundance of most mRNA transcripts was at least 1 order of magnitude below that value, which indicates that only a fraction of the population was actively expressing them at any given time. Estimates of mRNA transcript abundance were normalized by cell biovolume (i.e., transcripts per cubic micrometer, or [mRNA]) (Table S4), to discard potential indirect effects of cell size on transcript abundance in further analyses.

10.1128/mbio.03425-22.5 TABLE S2 Read counts obtained by HTSeq for each protein-coding gene in Prochlorococcus marinus MIT9301 in the samples collected during the long-term experimental acclimations. The sample names include the temperature, light/dark period, and the replicate number. Prochlorococcus marinus MIT9301 gene IDs, gene symbols, and annotated gene products according to the Bacterial and Viral Bioinformatics Resource Center (BV-BRC) are shown, as well as the corresponding RefSeq locus tags. Download Table S2, XLSX file, 0.5 MB. Copyright © 2023 Alonso-Sáez et al. 2023 Alonso-Sáez et al. https://creativecommons.org/licenses/by/4.0/ This content is distributed under the terms of the Creative Commons Attribution 4.0 International license.

10.1128/mbio.03425-22.6 TABLE S3 Estimates of mRNA transcripts per cell for each protein-coding gene in Prochlorococcus marinus MIT9301 obtained in the samples collected during the long-term experimental acclimations. The sample names include the temperature, light/dark period, and the replicate number. *P. marinus* MIT9301 gene IDs, gene symbols, and annotated gene products according to the Bacterial and Viral Bioinformatics Resource Center (BV-BRC) are shown, as well as the corresponding RefSeq locus tags. Download Table S3, XLSX file, 1.1 MB. Copyright © 2023 Alonso-Sáez et al. 2023 Alonso-Sáez et al. https://creativecommons.org/licenses/by/4.0/ This content is distributed under the terms of the Creative Commons Attribution 4.0 International license.

10.1128/mbio.03425-22.7 TABLE S4 Estimates of mRNA transcripts per biovolume (i.e., mRNA concentration) for each protein-coding gene in Prochlorococcus marinus MIT9301 obtained in the samples collected during the long-term experimental acclimations. The sample names include the temperature, light/dark period, and the replicate number. *P. marinus* MIT9301 gene IDs, gene symbols, and annotated gene products according to the Bacterial and Viral Bioinformatics Resource Center (BV-BRC) are shown, as well as the corresponding RefSeq locus tags. Download Table S4, XLSX file, 1.1 MB. Copyright © 2023 Alonso-Sáez et al. 2023 Alonso-Sáez et al. https://creativecommons.org/licenses/by/4.0/ This content is distributed under the terms of the Creative Commons Attribution 4.0 International license.

**FIG 2:**
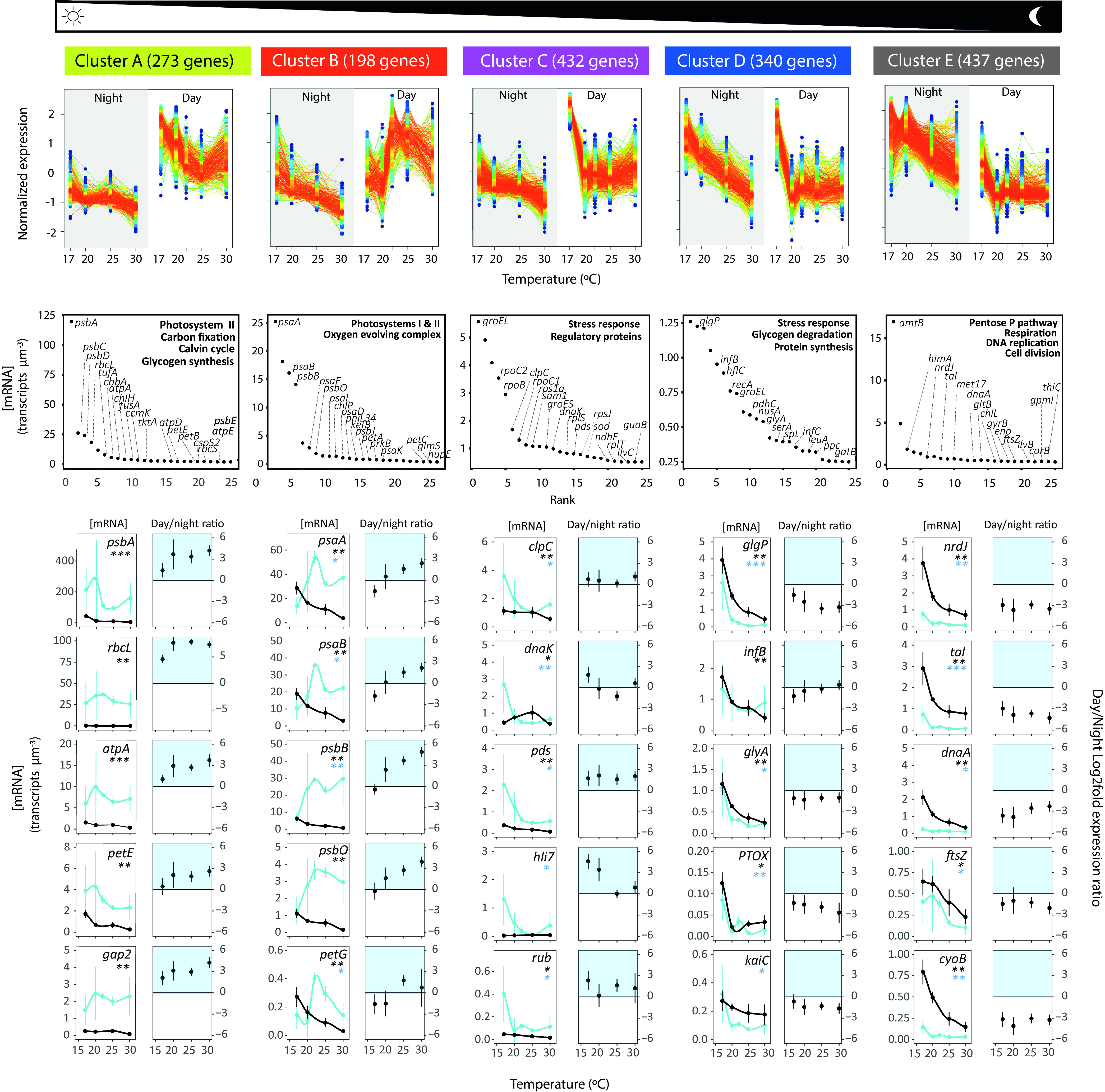
Clusters of Prochlorococcus marinus MIT9301 genes according to their pattern of daytime and nighttime expression along the thermal niche. Within each cluster, the right and left panels represent daytime and nighttime expression of the same genes, respectively. Dot colors indicate local density at each point of the scatterplot, with red circles indicating a high density of dots. Line colors within each SoftCluster indicate the membership value assigned by the fuzzy c-means soft clustering of each gene, ranging from 1 (red, high score) to 0.5 (blue, low score). Genes with a membership value lower than 0.5 are not plotted, and neither are they included in the total number of genes for each cluster (but they are included in Table S5). Below each cluster, the top 20 genes expressed are shown in rank order plots, and the expression levels (measured as [mRNA] and day/night log_2_ fold expression ratio) of a selection of representative genes are shown. Daytime values appear as blue lines and nighttime values as black lines. Between 3 and 7 biological replicate samples are represented, depending on the temperature treatment and error bars report the standard deviation between replicates. Asterisks in the plots denote significant differences in transcript concentration along the thermal gradient in daytime (blue asterisks) or nighttime (black asterisks) according to the Kruskal-Wallis test (*, *P*< 0.05; **, *P* < 0.01; ***, *P* < 0.001). In the log_2_ fold ratios, values above 0 represent preferential expression during daytime (highlighted in blue), while values below 0 represent preferential expression during nighttime.

**FIG 3:**
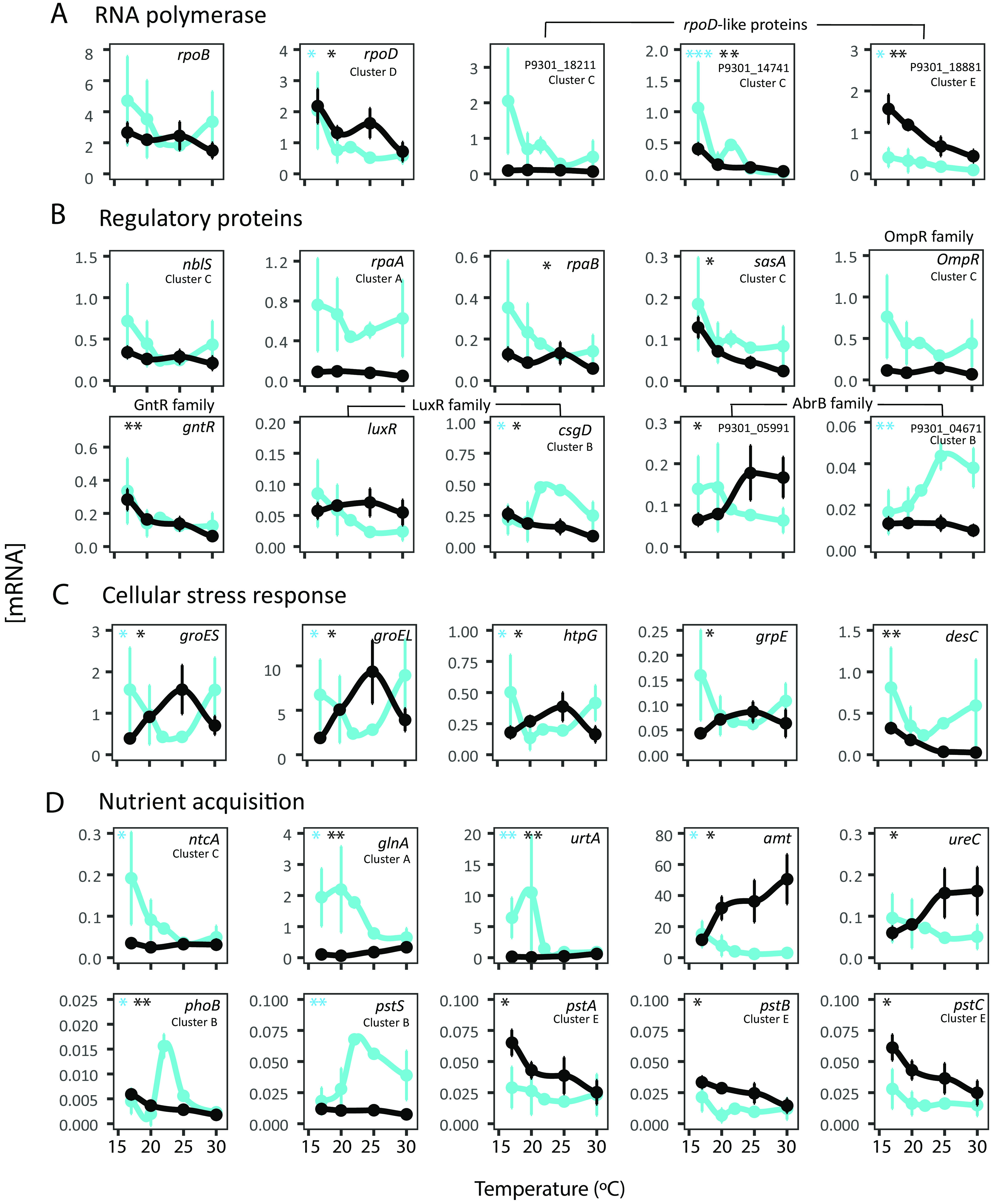
Cellular expression levels (measured as [mRNA]) in Prochlorococcus marinus MIT9301 during daytime (blue lines) and nighttime (black lines) along the thermal niche of (A) RNA polymerase components, including sigma factors, (B) histidine kinases and other regulatory proteins, (C) genes involved in the stress response, and (D) nitrogen and phosphate acquisition genes. Asterisks denote significant differences in transcript concentration along the thermal gradient in daytime (blue asterisks) or nighttime (black asterisks) according to the Kruskal-Wallis test (*, *P* < 0.05; **, *P* < 0.01; ***, *P* < 0.001). The SoftCluster membership of each gene is shown only for those cases where the probability score was >0.80.

A substantial fraction of MIT9301 genes were differentially expressed along the temperature gradient (i.e., exhibited significant variations in cellular [mRNA] estimates at different temperatures). In total, 61% of MIT9301 protein-coding genes were differentially expressed at nighttime, while this number was reduced to 30% during daytime (Kruskal-Wallis test; *P *< 0.05), in association with an increase in the variability of gene expression levels; while [mRNA] values of individual genes in different biological replicates were highly constrained at the T_opt_, a large variation was found during daytime at both thresholds of growth and particularly at the T_min_ (Fig. 2 and 3; Table S4). This suggests a reduced ability of MIT9301 cells to maintain a tight transcriptional control under challenging temperature conditions when cells are exposed to light. Notably, the expression of some sigma factors and key regulatory proteins from different families also showed this pattern (Fig. 3A and B).

10.1128/mbio.03425-22.8 TABLE S5 SoftCluster membership and probability scores for each of MIT9301 protein-coding genes. Download Table S5, XLSX file, 0.2 MB. Copyright © 2023 Alonso-Sáez et al. 2023 Alonso-Sáez et al. https://creativecommons.org/licenses/by/4.0/ This content is distributed under the terms of the Creative Commons Attribution 4.0 International license.

We identified five clusters of genes according to their patterns of daytime and nighttime expression at different temperatures (Fig. 2). More than 90% of MIT9301 protein-coding genes were assigned to one of these clusters with a probability score of >0.5 (Table S5), which indicates that these clusters were highly representative of the main thermal gene expression responses in this strain. Clusters A and E were represented by genes involved in core cellular and metabolic processes typically expressed in *Prochlorococcus* during daytime and nighttime, respectively (34). Clusters C and D were associated with mechanisms of cold stress response, as they were characterized by a strong upregulation at the T_min_ either during daytime or during both daytime and nighttime, respectively. Finally, the expression of genes in cluster B showed a decreasing trend from the T_opt_ toward the T_min_ during daytime (Fig. 2), paralleling the pattern observed in growth rates along the thermal niche (Fig. 1A). Therefore, we hypothesize that the expression of cluster B genes is associated with metabolic processes limiting the growth of *Prochlorococcus* when exposed to cold conditions.

Cluster A included genes related to C fixation and assimilation, such as RuBisCO (*rbcLS*), CO_2_ transporters (*csoS2*), carboxysome shell proteins (*ccmK*), the Calvin cycle (e.g., *gap2*, *tktA*, *glpX*, *pgk*, and *cbbA*), and glycogen synthesis (*glgABC*), consistently expressed during daytime along the thermal gradient (Fig. 2; Table S4 and Table S5). This cluster also included ATP synthesis genes (*atpADE*) and a few components of photosystem II (PS II) (*psbA*, *psbC*, and *psbD*). Cluster E included genes related to catabolic consumption (*cyoB* and *ndhD*), DNA replication (*dnaA*, *nrdJ*, and *gyrB*), cell division (*ftsZYQ*), and the pentose phosphate pathway (*tal*, *gnd*, and *zwf*), all of them upregulated at nighttime. Altogether, these essential pathways likely represent a transcriptional core, which Prochlorococcus marinus MIT9301 maintains under all temperature conditions.

Clusters C and D genes included different elements of the global stress response, such as cellular chaperones (*groES/groES*, *dnaK*, and *clpBCP*) (35, 36) and fatty acid desaturases (*desA* and *desC*), as well as mechanisms against oxidative damage, such as DNA repair (*recA* and *ruvB*) (37, 38), superoxide dismutase (*sod*), and the synthesis of antioxidant compounds like carotenoids (*pds* and *crtBH*) and rubredoxin (*rub*) (Fig. 2 and 3; Table S5). Notably, the expression of the chaperones *groEL*/*groES*, *grpE*, and *htpG* was strongly upregulated at the T_min_ only during daytime, suggesting a prioritization of their expression during the light-exposed period (Fig. 3C). Other metabolic processes upregulated at the T_min_ were the mobilization of energy storage (i.e., glycogen degradation, *glgP*), and the synthesis of proteins, as reflected by the increase in the [mRNA] of amino acid synthesis genes (*glyA*, *serA*, and *leuA*), translation initiation factors (*infABC*) and N acquisition genes (Fig. 2 and 3; Table S4).

The strong upregulation of N acquisition mechanisms at the T_min_ under light conditions was observed not only for genes predominately expressed during daytime at the T_opt_ (i.e., the global nitrogen regulatory protein gene *ntcA*, *glnA*, and urea transporter genes), but also for genes typically expressed at nighttime in *Prochlorococcus*, such as the ammonium transporter (*amt*) and urease (*ureABC*) genes (Fig. 3D; Table S4). This likely reflects the large cellular demand of N for protein synthesis during daytime under cold stress conditions. In the case of phosphate uptake, the high-affinity ABC transporter *pstABC* genes were upregulated under cold conditions at night, following the pattern of cluster E genes, possibly related to the cellular demand of P for DNA replication. In contrast, both copies of the periplasmic phosphate binding protein (*pstS*) showed maximum expression values around the T_opt_ during daytime, following the pattern of most photosynthetic genes (cluster B), which highlights the complexity of the thermal response of nutrient acquisition genes (Fig. 3D).

**FIG 4:**
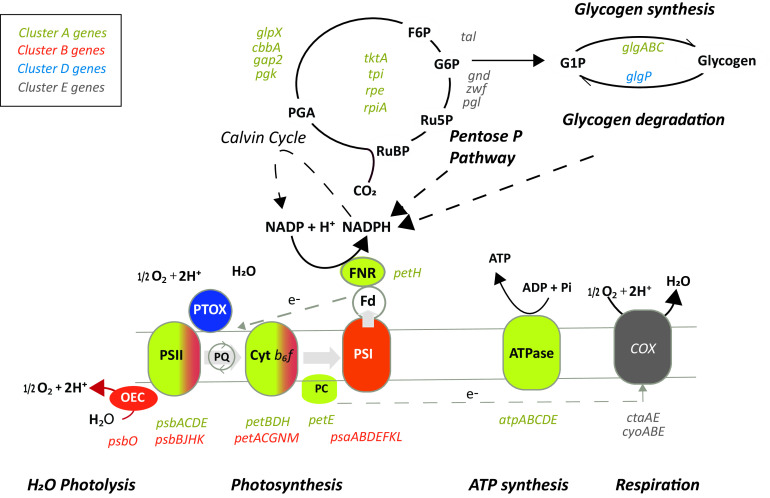
Schematic diagram showing some of the main components of the photosynthetic and carbon metabolism pathways in *Prochlorococcus*. Colors of genes (or their corresponding protein complexes) follow the same code as their respective SoftClusters in Fig. 2.

Finally, the expression of all components of the PS I complex (*psaABDEFKL*) and some of PS II (including *psbBJH* and the oxygen-evolving complex protein *psb*O) showed a gradual decrease in expression from a temperature close to the optimum to the T_min,_ (Fig. 2) in correlation with MIT9301 growth rates (Fig. 1). This expression pattern was different from those of other PSII components (*psbACD* [described above]), which were not differentially expressed during daytime along the thermal niche (Kruskall-Wallis; *P* > 0.05) (Fig. 2). Similarly, many components of the photosynthetic electron transport genes were assigned to either cluster B (*petACGNM*) or cluster A (*petBEDH*), implying a nonuniform transcriptional thermal response of all components of the photosynthetic apparatus (Fig. 4). Yet, a general pattern of upregulation of photosynthetic genes during nighttime under cold conditions was observed (Fig. 2), inducing changes in their day/night log_2_ fold expression ratio. This result suggests a loss in the capacity of cells to regulate their circadian expression under cold conditions. Accordingly, the day-night expression patterns of the circadian clock *kaiBC* genes were also impaired at the T_min_ (Fig. 2).

### Transcriptional response of photosynthetic genes in naturally occurring *Prochlorococcus* populations.

**FIG 5:**
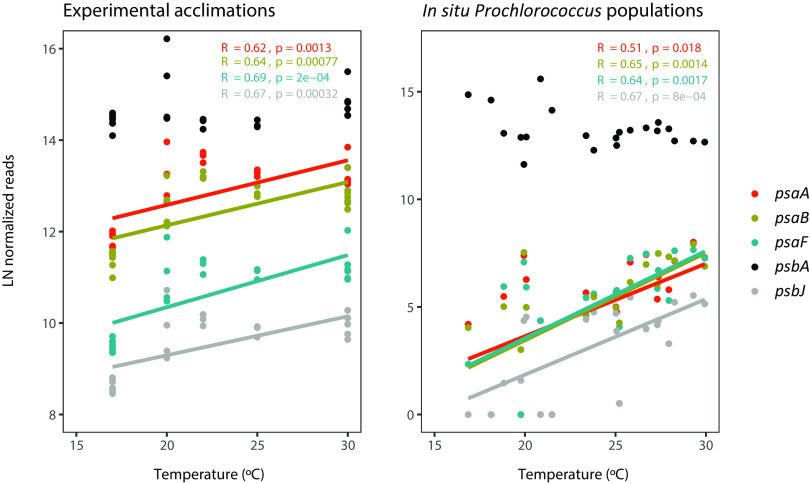
Expression patterns of a selection of *Prochlorococcus* photosynthetic genes of photosystem II (*psbA* and *psbJ*) and photosystem I (*psaA*, *psaB*, and *psaF*) along the thermal gradient 17 to 30°C in experimental acclimations (as analyzed in *P. marinus* MIT9301 by transcriptomics [left panel]) and *in situ* environmental conditions (as analyzed in reads mapping to MIT9301 identified in metatranscriptomes of the *Tara* Oceans data set [right panel]). In both cases, reads were normalized using DESeq2 and log transformed. Linear regression lines are shown for all genes with significant Spearman correlation coefficients at a *P* value of <0.05 (indicated in the plots).

After addressing the impact of thermal acclimation on MIT9301, we next aimed to test whether the transcriptional suppression of photosynthetic genes at the cold temperature observed experimentally was also found in natural *Prochlorococcus* populations *in situ*. For this, we identified reads closely related to MIT9301 in the *Tara* Oceans metatranscriptome data set (39), including samples distributed across different ocean basins along a comparable thermal gradient (Table S6). The number of reads mapping to MIT9301 (≥98% identity following our two-step filtering strategy [see Materials and Methods]) ranged from 6,379 to 9.62 million in the different environmental samples (Table S7). The DESeq2-normalized abundance of transcript counts from some of the most actively expressed genes of PS II (*psbA* and *psbD*) did not show any significant trend along the *in situ* thermal gradient from 17 to 30°C (Spearman correlation; *P* > 0.05), mirroring the response observed under culture conditions (Fig. 5; Fig. S2). In contrast, other PS II components (*psbJ*) were positively correlated with temperature in both the experimental and *in situ* data sets (Spearman *R* = 0.6; *P* < 0.01), indicating a downregulation at cold temperature (Fig. 5). Similarly, the downregulation of PS I genes at cold temperature was clearly observed both in the experimental and *in situ* data sets (Fig. 5), reinforcing the environmental relevance of this response. Temperature had the strongest correlation to photosynthetic transcript counts in comparison with all other tested environmental variables (Fig. S3), indicating that these correlations were not driven by covarying environmental parameters.

10.1128/mbio.03425-22.2 FIG S2 Expression patterns of Prochlorococcus marinus MIT9301 key photosynthetic genes (photosystem II in panel A and photosystem I in panel B) along the thermal gradient 17 to 30°C in experimental acclimations (as analyzed by transcriptomics [upper panels]) and under *in situ* environmental conditions (as analyzed by metatranscriptomics of the *Tara Oceans* data set [lower panels]). In both cases, reads were normalized using DESeq2 and log transformed. To visualize the trends, the linear regression lines are shown for all genes, but Spearman correlation coefficient values are only shown when slopes were significant at a *P* value of <0.1. Download FIG S2, PDF file, 0.3 MB. Copyright © 2023 Alonso-Sáez et al. 2023 Alonso-Sáez et al. https://creativecommons.org/licenses/by/4.0/ This content is distributed under the terms of the Creative Commons Attribution 4.0 International license.

10.1128/mbio.03425-22.3 FIG S3 Correlation between the DESeq2-normalized abundance of *psbA*, *psaB*, and *psaF* gene counts across the *Tara Oceans* metatranscriptomic data set and different environmental parameters. Spearman correlation coeﬃcients and *P* values are shown for each plot. Download FIG S3, PDF file, 0.4 MB. Copyright © 2023 Alonso-Sáez et al. 2023 Alonso-Sáez et al. https://creativecommons.org/licenses/by/4.0/ This content is distributed under the terms of the Creative Commons Attribution 4.0 International license.

10.1128/mbio.03425-22.9 TABLE S6 Basic information of the *Tara* Oceans metatranscriptomes selected for the analysis of expression of *Prochlorococcus* photosynthetic genes in natural surface samples, including Tara station ID, location, date and time of collection, depth, and *in situ* temperature. Download Table S6, XLSX file, 0.01 MB. Copyright © 2023 Alonso-Sáez et al. 2023 Alonso-Sáez et al. https://creativecommons.org/licenses/by/4.0/ This content is distributed under the terms of the Creative Commons Attribution 4.0 International license.

10.1128/mbio.03425-22.10 TABLE S7 Raw read counts obtained by HTSeq2 after alignment of a selection of *Tara* Oceans metatranscriptomes to the *Prochlorococcus* MIT9301 genome (at 98% identity). The metatranscriptomic samples have been identified by ENA sample ID. Download Table S7, XLSX file, 0.2 MB. Copyright © 2023 Alonso-Sáez et al. 2023 Alonso-Sáez et al. https://creativecommons.org/licenses/by/4.0/ This content is distributed under the terms of the Creative Commons Attribution 4.0 International license.

## DISCUSSION

There is a remarkable gap of fundamental knowledge of the molecular mechanisms of thermal acclimation in *Prochlorococcus*, as previous studies have concentrated on their cyanobacterial sister clade *Synechococcus* and eukaryotic phytoplankton (12–14, 20–22, 40, 41). Acquiring this knowledge is crucial for answering a long-standing question: what limits the ability of *Prochlorococcus* to expand to high-latitude environments? The decrease in the light-harvesting capacity of phytoplankton under cold conditions has been typically attributed to changes in the conformation of membrane lipids and proteins of their photosynthetic apparatus (14, 42). Our quantitative gene expression analysis shows that, in the case of *Prochlorococcus*, the inability to maintain the organism’s photosynthetic capacity under cold conditions is already critically compromised at the transcriptional level, as we found a clear suppression of the expression of most photosynthetic genes under cold conditions. The same pattern was found for reads mapping to MIT9301 in *in situ* metatranscriptomes across the global ocean, suggesting that this molecular mechanism may be relevant to explain the restricted distribution of *Prochlorococcus* to temperate zones. Notably, this response differs from those of previous studies targeting diatom cultures (26) or phytoplankton communities *in situ* (21, 39), indicating fundamental differences in the ability of *Prochlorococcus* to respond to cold temperature, which may be related to the organism’s exceptionally high sensitivity to oxidative stress (38, 43, 44).

Photosystems are naturally sensitive to light damage, which is exacerbated under low temperature (22). The cellular decision to shut down the sunlight energy conversion system under cold conditions likely arises from the inability of *Prochlorococcus* to cope with uncontrolled redox chemistry when there is an imbalance between the production of excited electrons by light and their metabolic consumption. The downregulation of photosystems would represent an emergency mechanism to slow down the electron flow and prevent the production of cell-damaging ROS (45, 46). Notably, the response was not equal for all photosystem components, as previously observed in cultured *Prochlorococcus* strains undergoing other types of stress, such as iron starvation (47), phage infection (48), or high light exposure (49). On the one hand, the high expression of *psbA* transcripts under all temperature conditions is likely related to the need to maintain an exceptionally high turnover rate of this protein (50), being under a different regulatory regime from other PS components (34, 51, 52). On the other hand, the selective downregulation of PS I probably arises from the strong need to protect PS I from oxidative stress, as this photosystem typically lacks efficient repair machinery, and therefore its damage may be practically irreversible (53). Interestingly, MIT9301 also upregulated the plastoquinol terminal oxidase (PTOX) at the T_min_ (Fig. 2), which is thought to function as a safety valve in cyanobacteria to avoid electron flow toward PS I (54).

In addition to the transcriptional suppression of photosynthetic genes, we found that under light conditions, cold temperature induced a loss in the regulatory capacity of MIT9301 cells (Fig. 2). This was reflected in the loss of the circadian day/night gene expression ratio and the increased variability in the concentration of cellular mRNA transcripts among biological replicates. In a previous study, it was suggested that higher levels of stochasticity in *Prochlorococcus* gene expression during the transition from photosynthesis to the use of internal energy is related to the accumulation of ROS (55). Our transcriptomic results are consistent with the idea of oxidative stress impacting the regulatory capacity of *Prochlorococcus*, as we found the upregulation of different genes against this source of stress under cold conditions (Fig. 2 and 3). Notably, at the T_min_ we observed a high variability in the expression of regulatory proteins that modulate the cellular response to daily light fluctuations in cyanobacteria, including sigma factors (*rpoD*) (56, 57), the NblS-RpaB two-component system (58), and the SasA-RpaA clock output system (59, 60) (Fig. 3). Assuming that mRNA transcript levels reflect the protein levels of these regulators, our results would imply a critical impairment of their coregulated networks. Other mechanisms of transcriptional regulation in *Prochlorococcus* (i.e., RNA-based regulatory strategies) (61) were likely also compromised at low temperature, as evidenced by the upregulation of the RNase *rne* gene at the T_min_ (see Table S4 in the supplemental material).

Marked changes in the day/night gene expression ratio of some of *Prochlorococcus* key functional genes along the thermal niche imply a deregulation of one of their fundamental features: i.e., the coordination of transcriptional oscillations with the daily light/dark cycle. This feature is key to optimize cellular processes through anticipating and synchronizing transcription of photosynthetic genes with daylight hours (34, 62, 63). At T_opt_, the day/night expression preferenda of most key functional genes of MIT9301 matched those previously observed in the model strain MED4 (34), reinforcing the idea that maintaining this transcriptional choreography is a highly conserved and critical trait. The deregulation observed at the cold temperature led to the paradoxical situation where, after subjective sunrise, chilled *Prochlorococcus* MIT9301 cells contained severalfold less PS I transcripts than after subjective sunset, which supports the idea of a malfunctioning regulatory network (Fig. 2). With only two samplings over the daily cycle, we cannot ensure whether the observed changes were due to random misregulations or variations in the phasing or amplitude of their diel gene expression patterns. Yet, considering that we were sampling the two daily periods when most *Prochlorococcus* genes show maxima in expression (34), the inversion of their day/night preferenda is remarkable and likely has an important impact on *Prochlorococcus* fitness.

In contrast to the pattern observed in day-time samples, genes related to *Prochlorococcus* cell division cycle, typically expressed during nighttime, showed rather constrained [mRNA] values between replicates. We observed an increase in [mRNA] of those genes toward cold conditions, which may be related to the predominant cell cycle phase of the cells. As the timing of cell division is delayed in Prochlorococcus marinus MIT9301 when acclimated to cold temperature (64), our results likely reflect a situation where a higher proportion of cells were still undergoing the S phase at 17°C and 20°C at the time of sampling (i.e., 3 h after subjective dusk) compared to other acclimation temperatures.

At the warm temperature threshold, the upregulation of a variety of cellular chaperones (e.g., *groEL/groES*, *htpG*, and *grpE*) reflected that cells were undergoing substantial stress. However, no drastic changes were generally observed in the expression of most protein-coding genes at the T_max_. Accordingly, a previous study on the short-term response of MED4 to warm stress found that the synthesis of new polypeptides was not induced (65). We had previously shown that *Prochlorococcus* MIT9301 cells decrease in size after long-term warm acclimation, in accordance with the temperature-size rule (64). Interestingly, here, we found a conspicuous streamlining in the transcript inventory of cells in parallel with the progressive reduction in cell size (Fig. 1). While at nonrestricting growth temperature, the average estimate of mRNA transcript abundance per cell was within the range reported for environmental marine bacteria (i.e., ca. 85 to 255 transcripts per cell) (32), under warm conditions this average dropped down to 30 mRNA transcripts of protein-coding genes per cell at nighttime. At increasing temperatures, cells may require a lower mRNA content to achieve the same translation rates, as suggested for rRNA content in the translation-compensation hypothesis (66). Yet, it should be considered that cellular reactions involving small numbers of molecules are intrinsically noisy, being dominated by fluctuations in concentration and stochasticity (67, 68). Thus, the decline in the transcript inventory under warm conditions also invites the hypothesis that there is a critical threshold in the number of mRNA copies beyond which cells lose regulatory capacity, contributing to the growth arrest.

In summary, previous studies on phytoplankton have identified the cellular membrane and translational apparatus as central elements associated with the thermal adaptation at the transcriptional level (21, 26). Some of these mechanisms were also observed in MIT9301, such as the cold upregulation of the translational machinery, likely to compensate for the general slowdown in protein synthesis rates, and fatty acid desaturases critical for maintaining the fluidity of cellular membranes (24). Yet, we found that in the case of *Prochlorococcus*, a general downregulation of photosystem components and impairment in their circadian expression patterns emerged as major features potentially impacting their physiology when approaching cold conditions. Low temperature has been previously shown to nullify the circadian rhythm in different organisms, including plants and different phytoplankton species, moving the circadian oscillation to a damped oscillation (see references in reference 27). *Prochlorococcus* contains only a minimalist circadian system; thus, its cell cycle is likely more easily perturbed than that from organisms containing a complete *kaiABC* gene set. As oxidative stress relates to both the need to protect the photosynthetic machinery and the ability of *Prochlorococcus* species to “reset” their daily cycles in the morning hours (69), it is plausible to link this source of stress to the molecular response observed, but experimental evidence would be required to confirm this point.

The disruption of the transcriptional choreography in *Prochlorococcus* may be a differentiating factor compared to *Synechococcus* and eukaryotic phytoplankton, which do not require maintenance of such a fine-tuned daily expression rhythm to maintain their metabolism. The higher tolerance of *Synechococcus* species to oxidative stress (38) may also imply a more resilient photochemistry when exposed to cold conditions, enabling them to colonize higher latitudes. Additionally, we postulate that increased levels of stochasticity in gene expression under challenging temperature conditions may contribute to a decrease in *Prochlorococcus* fitness. These remain as interesting hypotheses to test in future studies, with implications for the functioning of this global primary producer and, thus, marine carbon biogeochemistry under future oceanic conditions.

## MATERIALS AND METHODS

### Growth of cultures and temperature acclimation.

We grew nonaxenic cultures of Prochlorococcus marinus MIT9301 obtained from the Roscoff Culture Collection (RCC) in PCR-S11 culture medium (70), based on Red Sea salt (Houston, TX, USA). Cultures were grown under a 12-h/12-h photoperiod and irradiance of 120 μmol quanta m^−2^ s^−1^ in polycarbonate flasks with vented caps. During the acclimation, we maintained the cultures in exponential growth by performing serial transfers before cell density reached 30% of the maximum yield. The acclimation started from 22°C (i.e., temperature of maintenance in RCC), and temperature was progressively changed toward the upper and lower thermal thresholds. At each acclimation step, the temperature was changed by a maximum of 2°C and down to 0.2°C when approaching the temperature thresholds, to avoid lethal thermal stress (see Fig. S1 in the supplemental material). We considered that the culture had been fully acclimated to a temperature treatment when the growth rate stayed stable in at least two consecutive growth curves after at least 8 generations. Flow cytometry was used for sustained monitoring of the culture growth during the acclimation process. Samples were fixed with glutaraldehyde (final concentration of 0.025%) for 10 min at room temperature and under dark conditions and frozen at −80°C until analysis in a FACSCalibur flow cytometer (Becton Dickinson). Estimates of cell diameter were obtained based on the natural logarithmic-transformed side scatter (SSC) using the calibration provided in reference 71. Cell volumes were calculated from the resulting estimates of cell diameter, assuming a spherical cell shape.

### RNA sample collection and extraction.

Once the cultures reached full acclimation to the temperatures selected (17, 20, 22, 25, and 30°C), we reinoculated biological replicate batch cultures into fresh medium (160 mL) and collected samples for RNA-Seq analysis during exponential growth (with cell density values ranging between 6 × 10^7^ and 17 × 10^7^ cells mL^−1^) (see Table S1 in the supplemental material). For each temperature and biological replicate (ranging from 3 to 7, depending on the experimental treatment) (Table S1), samples for RNA extraction were collected 3 h after the onset and offset of the photoperiod (daytime and nighttime, respectively). Exceptionally, for the acclimation temperature 22°C, only daytime RNA samples were available. Samples were filtered on 0.22-μm-pore-size polyethersulfone (PES) filters using a vacuum pump at a pressure of 5 lb/in^2^. Immediately after filtration, filters were snap-frozen in liquid nitrogen and stored at −80°C. The time elapsed from the start of the filtration until freezing was always less than 2 min.

Quantitative benchmarked RNA extraction was performed following reference 33 using five RNA standards from Saccharolobus solfataricus (formerly Sulfolobus solfataricus) P2 (NCBI Taxon ID 273057) obtained by *in vitro* transcription of genomic templates of the isolate (standards 3, 6, 7, 13, and 14, as described in reference 33). The filters were spiked with the RNA standards individually (10 to 28 μL) at a concentration of ca. 20 pg μL^−1^ prior to the initiation of the RNA extraction. Subsequently, RNA samples were extracted with the mirVana kit (Ambion), and DNA was removed using the TURBO DNase (Ambion). Samples were depleted from rRNA using the Ribo-Zero rRNA removal kit (Bacteria; Illumina), and a quality check was performed using a Bioanalyzer (Agilent). mRNA samples were concentrated using the Zymo Clean & Concentrator kit, and cDNA libraries were constructed using the TruSeq stranded mRNA sample preparation kit (Illumina). cDNA libraries were sequenced as 75-bp paired-end reads on an Illumina HiSeq v4 platform (CNAG, Spain).

### Quantitative gene expression analysis of *Prochlorococcus* MIT9301 under culture conditions.

The sequence read quality check was performed with the FastQC tool (72), and Trimmomatic (73) was used to trim raw sequences (SLIDINGWINDOW:50:35 and MINLEN:50) and pair those passing quality thresholds. rRNA sequences were removed using SortMeRNA (74), and the remaining reads were mapped with Bowtie2 (75) (using the “–non-deterministic” parameter) against the Prochlorococcus marinus MIT9301 genome (NCBI Taxon ID 167546). The same procedure was done with the S. solfataricus genome to identify RNA internal standard reads. Read count tables were obtained using HTSeq (76) with the following parameters: “–stranded = reverse -a 10 -m intersection-nonempty.” Quantitative estimates of individual transcript abundance (Ta) of MIT9301 protein-coding genes in each RNA sample were obtained following the calculations described in (77): Ta = (Ts × Sa)/Ss. Ta corresponds to the estimated number of transcripts of an individual MIT9301 protein-coding gene, Ts corresponds to the number of reads assigned to the corresponding MIT9301 protein-coding gene, Sa corresponds to the molecules of internal RNA standards spiked to the RNA sample, and Ss corresponds to the number of reads assigned to S. solfataricus internal standards. In this calculation, one of the standards (standard 14) was removed from the analysis because it was consistently recovered in higher proportion than other standards. Ta values were divided by total cell abundance or estimates of total cell biovolume collected in the corresponding RNA filter to obtain estimates of transcript abundance per cell and per volume (i.e., transcript concentration), respectively (Table S1 to Table S3). This normalization is relevant because cell size, which varies over the thermal gradient in this strain (Fig. 1A), can impact the number of cellular transcripts (78, 79).

### Bioinformatic analysis of transcriptional patterns of *Prochlorococcus* photosynthetic genes in oceanic samples and culture conditions.

For comparison of the expression patterns of *Prochlorococcus* photosynthetic genes along the same thermal gradient under environmental and culturing conditions, two data sets were produced using a common normalization method (DESeq2) (80). The experimental data set was obtained from the raw read counts obtained by HTSeq2 in the long-term thermal acclimation experiments, as explained above. To obtain the environmental database, a data set of *Tara* Oceans metatranscriptomes (39) was initially selected based on the latitude range where *Prochlorococcus* is found in the ocean (i.e., between 45°N and S), the thermal range of MIT9301 (17°C to 30°C), and the time of sample collection (between 6 a.m. and 12 p.m., when the expression of photosynthetic genes should be close to their maximum). Fastq sequence files were quality filtered with fastp (81) using default parameters, and rRNA sequences were removed with RiboDetector (82) with the option “–ensure” selected. The remaining reads were filtered to remove reads not affiliated with *Prochlorococcus*, using a custom-made database with the complete genome sequences in the MarRef database, which included 16 *Prochlorococcus* and 17 *Synechococcus* genomes (83), and eukaryotic genes of interest extracted from Refseq (84): i.e., *psaA*, *psaB*, *psaD*, *psaF*, *psaL*, *psbA*, *psbD*, and *psbO*, searched with Entrez filter [Gene Name] AND ((protists[filter] OR plants[filter]) AND refseq[filter]). Metatranscriptome sequences closest to *Prochlorococcus* were identified based on blastn v.2.12.0+ searches (identity of ≥98% and bitscore of ≥30), and only those with the highest bitscore to *Prochlorococcus* sequences were considered further. Subsequently, we applied a two-step filtering strategy to obtain read counts closely related to MIT9301. First, we aligned each metatranscriptome sample against the MIT9301 genome using BWA mem v.0.7.17-r1188 with default parameters (85). Next, we discarded alignments that had a percentage of identity lower than 98%, a read length lower than 50 bases, and a number of matches lower than 50% of the total length of each aligned sequence. The percentage of identity was computed as 100 × [N_m/(N_m + N_i)], where N_m corresponds to the number of matches in the alignment and N_i to the number of mismatches. The number of matches was obtained by parsing the MD tag of the alignment record, while the number of matches and mismatches was obtained from the CIGAR string in the SAM file. Once alignments were filtered, we proceeded to count aligned reads with HTSeq v.2.0 with default parameters (86). Next, we removed noncoding genes from the count matrices as well as genes that had no counts across all conditions, to facilitate the consequent normalization. We also discarded samples that had counts of less than 10 genes other than the *psbA* gene. Finally, we normalized count data using the default DESeq2 v.3.15 count normalization workflow (80). All analyses were assisted with customized Python code (87) available at https://github.com/Robaina/prochlorococcus (88).

### SoftClustering and statistical analyses.

Clusters of differentially expressed genes that responded similarly over the thermal gradient were identified using SoftClustering following reference 89. A matrix of *n* genes × 9 treatments (4 temperatures at nighttime and 5 temperatures at daytime) was used as input data. Data for each gene were standardized to zero mean and unit variance. The optimal value of the parameter *m* in the Mfuzz algorithm was estimated through randomization following reference 90. The number of clusters was chosen to maximize the functional enrichment of gene clusters (COGs) and the ClusterJudge method (91). The standardized data were clustered by a generalized version of the Fuzzy c-means algorithm. Finally, statistically significant differences in cellular transcript concentration among temperature regimes were determined by the Kruskall-Wallis test using R.

### Data availability.

Raw reads have been submitted to ENA under project no. PRJEB54738 (accession no. ERS12467859 to ERS12467904).
