# Supplementary material for: Transcriptional Mechanisms of Thermal Acclimation in Prochlorococcus
Source: mBio. 2023 Apr 13;14(3):e03425-22. doi: 10.1128/mbio.03425-22 (PMC10294614; doi:10.1128/mbio.03425-22)
Supplement: FIG S1 [file mbio.03425-22-s0001.pdf]

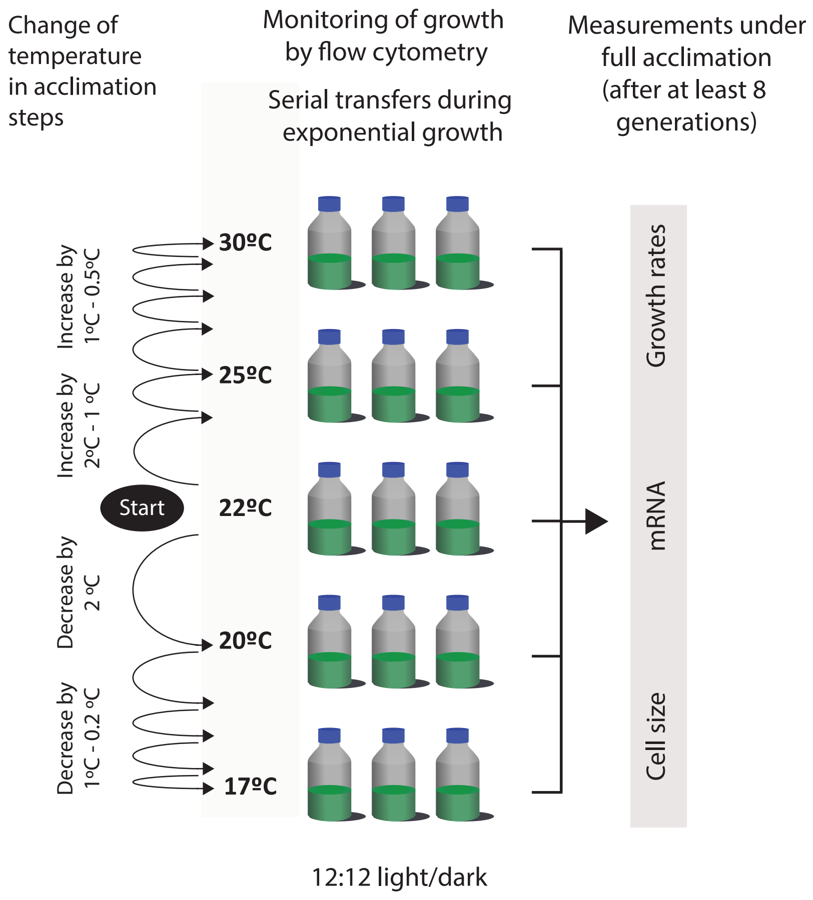

Figure S1. Schematic diagram showing the experimental approach and sampling strategy during the long-term thermal acclimation experiments. While only 3 biological replicates are shown, the real number of replicates varied betwen 3 and 7 depending on the temperature treatment.
