# Supplementary material for: Transcriptional Mechanisms of Thermal Acclimation in Prochlorococcus
Source: mBio. 2023 Apr 13;14(3):e03425-22. doi: 10.1128/mbio.03425-22 (PMC10294614; doi:10.1128/mbio.03425-22)
Supplement: FIG S2 [file mbio.03425-22-s0002.pdf]

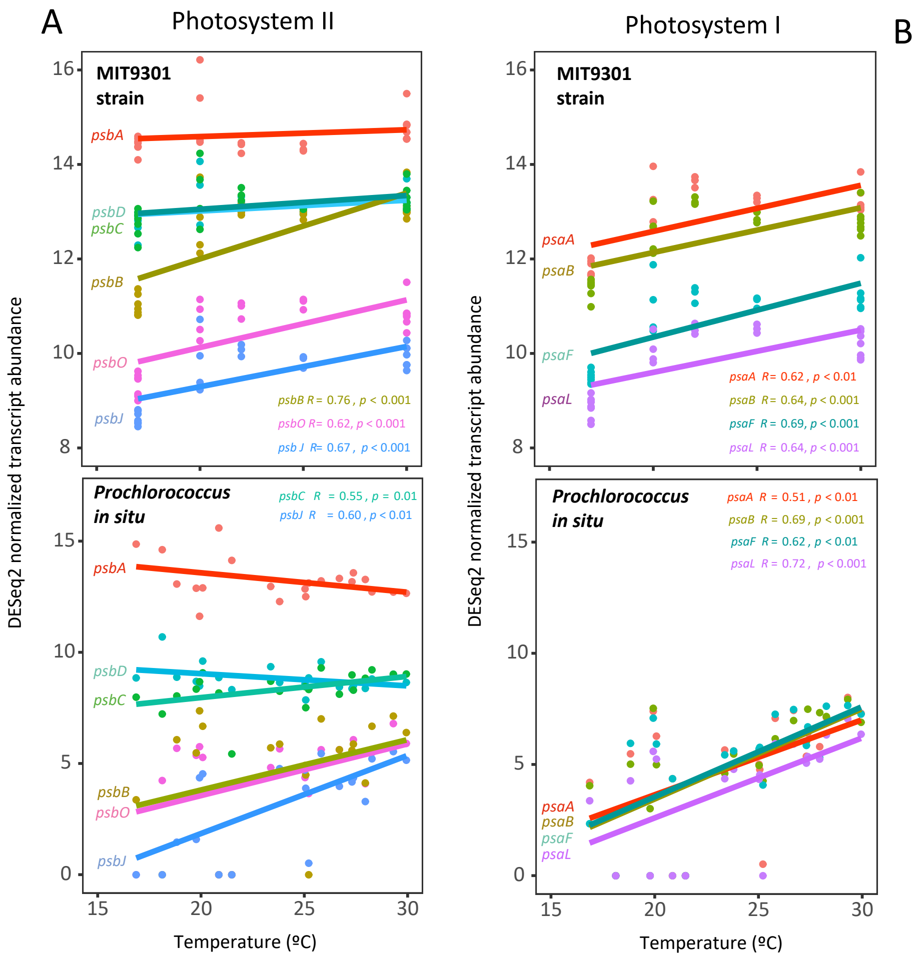

Figure S2. Expression patterns of *Prochlorococcus marinus* MIT9301 key photosynthetic genes (Photosystem II in A and Photosystem I in B) along the thermal gradient 17 to 30ºC in experimental acclimations (as analysed by transcriptomics, upper panels), and *in situ* environmental conditions (as analysed by metatranscriptomics of the *Tara Oceans* dataset, lower panels). In both cases, reads were normalized using DESeq2 and log-normalized. To visualize the trends, the linear regression lines are shown for all genes, but Spearman correlation coefficient values are only shown when slopes were significant at a *p*-value < 0.1.
