# Supplementary material for: Transcriptional Mechanisms of Thermal Acclimation in Prochlorococcus
Source: mBio. 2023 Apr 13;14(3):e03425-22. doi: 10.1128/mbio.03425-22 (PMC10294614; doi:10.1128/mbio.03425-22)
Supplement: FIG S3 [file mbio.03425-22-s0003.pdf]

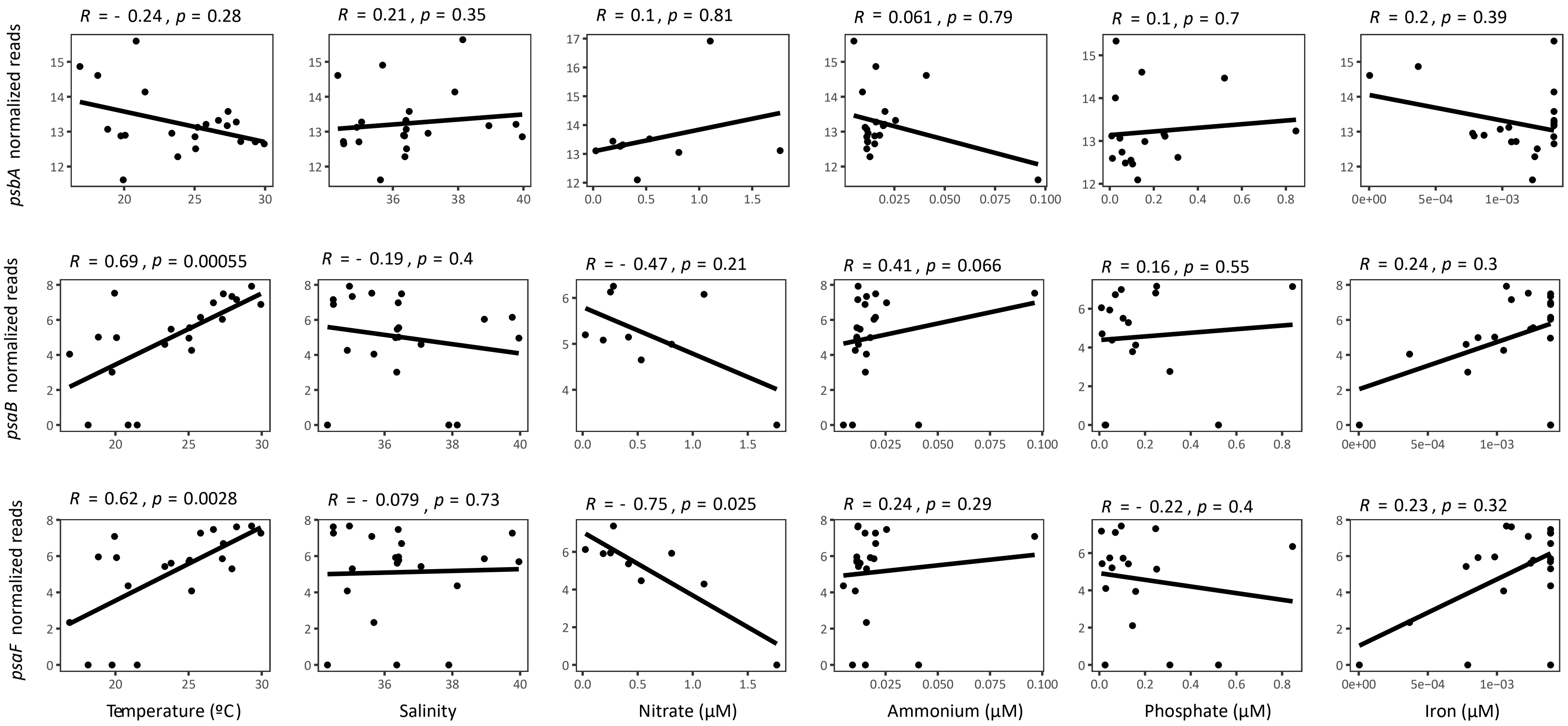


**Figure S3**. Correlation between the Deseq2 normalized abundance of *psbA*, *psaB* and *psaF* gene counts across the *Tara Oceans* metatranscriptomic dataset and different environmental parameters. Spearman correlation coefficients and *p*-values are shown for each plot.
